# The protective effect of isoniazid preventive therapy on tuberculosis incidence among HIV positive patients receiving ART in Ethiopian settings: a meta-analysis

**DOI:** 10.1186/s12879-019-4031-2

**Published:** 2019-05-10

**Authors:** Demeke Geremew, Aklilu Endalamaw, Markos Negash, Setegn Eshetie, Belay Tessema

**Affiliations:** 1 Department of Medical Immunology and Molecular Biology, School of Biomedical and Laboratory Sciences, University of Gondar, Gondar, Ethiopia; 2 Department of Pediatrics and Child Health Nursing, School of Nursing, College of Medicine and Health Sciences, University of Gondar, Gondar, Ethiopia; 3 Department of Medical Microbiology, School of Biomedical and Laboratory Sciences, University of Gondar, Gondar, Ethiopia

**Keywords:** Tuberculosis, HIV, ART, Isoniazid preventive therapy, Meta-analysis

## Abstract

**Background:**

Tuberculosis (TB) and HIV makeup a deadly synergy of infectious disease, and the combined effect is apparent in resource limited countries like Ethiopia. Previous studies have demonstrated inconsistent results about the protective effect of isoniazid preventive therapy (IPT) on active TB incidence among HIV positive patients receiving ART. Therefore, the aim of this meta-analysis was, first, to determine the protective effect of IPT on active tuberculosis incidence, and second, to assess the pooled incidence of active TB among HIV positive patients taking ART with and without IPT intervention in Ethiopia.

**Methods:**

PubMed, Google scholar and Cochran library databases were searched from April 1 to 30, 2018. Two independent authors explored and assessed studies for eligibility, and extracted data based on predefined criteria. Studies that reported TB incidence among HIV positive patients taking ART in Ethiopia with and without IPT concomitant intervention, and with a clear stratified data on the incidence of TB based on the duration of IPT intervention were selected. A random effects model was used to estimate risk ratios and the pooled incident TB with the respective 95% confidence intervals.

**Results:**

We identified 7 suitable studies in this analysis. Accordingly, IPT reduced the risk of TB incidence by 74%, risk ratio (RR) 0.26 (95% CI; 0.16–0.43%), compared to no IPT group. Moreover, IPT for 12 months reduced incident TB by 91% (RR: 0.09, 95% CI: 0.04 to 0.21), whereas 6 months IPT averted TB incidence by 63% (RR: 0.37, 95% CI: 0.26 to 0.52). The overall pooled incident TB among HIV infected patients receiving ART was 10.30% (95% CI; 7.57–13.02%). Specifically, incident TB among study cohorts with and without IPT was 3.79% (95% CI; 2.03–5.55%) and 16.32% (95% CI; 11.57–21.06%) respectively.

**Conclusion:**

IPT reduced the risk of incident TB among HIV positive patients receiving ART in Ethiopian settings. Moreover, the duration of IPT intervention has effect on its protective role. Thus, scaling up the isoniazid preventive therapy program and its strict compliance is necessary to avert HIV fueled tuberculosis.

**Study protocol registration:**

CRD42018090804.

**Electronic supplementary material:**

The online version of this article (10.1186/s12879-019-4031-2) contains supplementary material, which is available to authorized users.

## Background

Tuberculosis (TB) remains a main public health problem throughout the world, even with the recent advancement of extensive global TB control efforts [1]. According to the World Health Organization (WHO) 2017 report, there are 10.4 million incident cases of TB, out of which an estimated 1.04 million (10%) occurred in HIV positive patients. The risk of developing active TB in people living with HIV (PLWH) is 21 times higher than the rest of the world population [2]. Thus, it was clear that HIV infection is one of the risk factors for developing active TB due to altered immune state associated with low CD4^+^ T lymphocyte counts of HIV positive patients [3]. Most of HIV associated tuberculosis cases (79%) are from African followed by the Southeast Asia region which accounted for 11% of the total cases [4].

Although antiretroviral therapy (ART) is the single most significant way to reduce incident TB in PLWH, HIV positive patients receiving ART remain very susceptible to TB. Hence, TB remains the leading cause of death even among HIV infected patients receiving ART [5]. Therefore, besides early ART initiation, isoniazid preventive therapy (IPT) is the key intervention to prevent TB among PLWH. Moreover, WHO also reported that IPT taken for 6–12 months has reduced the risk of TB by 33% among all people with HIV and by 64% in HIV infected patients who have a positive tuberculin skin test (TST) [5]. Regarding the concomitant use of IPT with ART, recent evidence revealed that the combined use of IPT and ART have synergetic effect in averting incident TB among HIV positive patients [6]. Despite promising recent progresses of IPT in reducing incident TB among HIV infected patients significantly, it is not commonly implemented [7].

In Ethiopia, IPT provision for HIV positive patients is suggested by the national TB/HIV collaborative activities guideline and is implemented since 2007 [8]. However, its implementation has faced a lot of challenges in Ethiopia including; poor patient adherence, poor patient empowerment and proper counseling on IPT [9], fear of side effects and developing isoniazid (INH) resistant TB, and as well as lack of commitment of health managers to scale up the program [10]. Moreover, the pooled effectiveness of IPT in preventing incident TB among HIV infected patients receiving ART has not been assessed yet in Ethiopia. Therefore, the purpose of this review was, first, to assess the effectiveness of IPT in averting active TB incidence, and second, to determine the pooled incident TB among HIV positive patients taking ART with and without IPT in Ethiopia.

## Methods

### Study settings

Although the first evidence of HIV epidemics in Ethiopia was reported in 1984, free ART service was launched in January 2005, and implemented by public hospitals in March 2005 [11]. Moreover, access to ART service was increased rapidly in Ethiopia after HIV care decentralization was introduced in 2006 [12]. Now, ART service is freely available in 913 health facilities of which 765 are health centers, and the rest are hospitals [11]. Though ART can reduce incident TB in HIV positive patients, HIV fueled tuberculosis is still resurging in Ethiopia. Therefore, IPT was adopted to reduce incident TB as per the WHO treatment for HIV guidelines and provided for HIV infected patients in Ethiopia [8].

### Definitions

The following terms were used in this meta-analysis; incident TB described as all new TB cases from day zero to the end of the last day of observation. Incident TB was calculated by dividing the number of HIV infected patients receiving ART with TB disease by the total number of study subjects at risk at the start of observation, and multiply by 100. Pooled incident TB defined as an estimate calculated from reported TB incidences from included studies. However, this proportion does not adjust for whether the study cohorts received 6 or 12 months of IPT.

### Reporting and study protocol registration

This review was conducted based on Preferred Reporting Items for Systematic Reviews and Meta-Analyses (PRISMA) statement guideline [13] (Additional file 1). The study has been registered in International Prospective Register of Systematic Reviews (PROSPERO) database with protocol number, CRD42018090804.

### Types of studies

All studies considered in the meta-analysis were cohort studies comparing the effect of IPT with those of no intervention on active TB incidence among HIV positive patients taking ART in Ethiopia. The Participants, Interventions, Comparisons, and Outcomes (PICOs) were considered to identify studies and extract data for this review.

### Types of participants

All HIV positive patients receiving ART and diagnosed with active incident TB were eligible for inclusion in this study.

### Types of intervention

The type of intervention was IPT at least for 6 months follow up to determine the incidence of active TB.

### Comparison

No intervention group was considered as a comparison group. The study included IPT versus no IPT intervention group. We excluded IPT versus no IPT intervention when reported in pre-ART HIV infected patients.

### Outcomes of interest

The outcome was active TB incidence among HIV positive patients taking ART. Thus, the primary outcome of interest was to measure the risk ratio of developing active TB among HIV infected patients receiving ART with IPT compared to without IPT intervention group. Moreover, the results of IPT intervention has been stratified by duration of INH treatment (6 months versus 12 months). Secondly, we have also assessed the incidence of active TB among HIV positive patients taking ART with and without IPT intervention.

### Search strategy for identification of studies

We made a comprehensive literature search using PubMed, Google scholar and Cochran library databases from April 1 to 30, 2018. The PICO (population, intervention, comparison and outcomes) search terms used were: [“HIV” OR “HIV patients” AND “ART” OR “HAART” AND “Isoniazid preventive therapy” OR “Isoniazid prophylaxis” AND “no isoniazid prophylaxis” OR “placebo treatment” AND “TB infection” OR “TB incidence” AND “Ethiopia”]. Besides, reference lists of relevant articles were also searched to find additional studies. We communicated the authors of included studies whenever information to be extracted from the article needs clarification.

### Inclusion and exclusion criteria

All original research articles conducted only in Ethiopian settings that fulfill the following criteria were included in this meta-analysis. Articles with cohort study design, studies reporting TB incidence among HIV positive patients taking ART with and without IPT concomitant intervention. Besides, articles with a clear stratified data on the incidence of TB based on the duration of IPT intervention were included. Lastly, studies published in English were considered in this analysis.

However, review articles, conference abstracts, editorials, proceedings, case reports, studies without full-text access, and studies that reported incident TB in HIV positive cases on pre-ART with IPT intervention were excluded. Likewise, studies that reported prevalence of TB in HIV positive cases who were on ART with and without concomitant IPT intervention were also excluded.

### Study selection and data extraction

Two independent reviewers, DG and SE examined the retrieved studies for inclusion. Discrepancy between the two authors was resolved by discussion. If the disagreement could not be fixed, a third author (AE) was involved to inspect the article and resolve the discrepancy. In the meantime, reasons for excluded studies were acknowledged.

Data abstraction format was built and pilot tested with a representative sample of studies to be reviewed, then it was summarized using a table. Data were extracted and entered into a table using Microsoft Excel spreadsheet and exported into Stata version 11.0 (StataCorp, College Station, TX, USA) for further analysis. Two authors independently extracted data from included studies. In case of discrepancy, a third author extracted data and the authors of original studies were contacted whenever additional information was necessary. The following descriptive information was extracted from each study: study author and year of publication, study design, study period, participants’ age, sample size and number of participants with the outcome, number of participants with and without intervention and the outcome, laboratory methods employed to diagnose TB, duration of IPT follow up and intervention outcome.

### Measures of treatment effect

The effect of IPT on active TB incidence among HIV positive patients receiving ART was determined by calculating the risk ratio (RR) and 95% confidence intervals (CIs) for dichotomous data. Moreover, the pooled incident TB with 95% CIs for HIV infected patients with and without IPT was calculated by dividing new TB cases with total sample size in each category.

### Risk of bias and quality assessment

Two independent authors evaluated the risk of bias in the included studies. Assessment was done based on the Cochran Hand Book of Systematic Reviews of Interventions [14]. Besides, the quality of included studies was evaluated by using the Newcastle-Ottawa quality assessment scale for cohort studies [15]. Thus, the following items were used to appraise the reports: 1) representativeness of the exposed cohort, 2) selection of the none exposed cohort, 3) ascertainment of exposure, 4) demonstration that outcome of interest was not present at start of study, 5) comparability of cohorts on the basis of the design or analysis, 6) assessment of the outcome, 7) was follow up long enough for outcomes to occur, 8) adequacy of follow up of cohorts. Based on the Newcastle-Ottawa quality assessment scale, all included studies have low risk of bias for methodological quality (Additional file 2).

### Statistical analysis

Stata version 11.0 software was used for statistical data analysis. The random effects models (DerSimonian-Laird method) [16] were used to calculate RR for dichotomous variables and its corresponding 95% CIs. In addition, it was also employed to pool the aggregate results of all included studies. Besides, we also used Freeman Tukey arcsine methodology to address stabilizing variances [17]. The standard approach of inverse variance method to calculate pooled estimates and standard errors does not perform well in meta-analysis of prevalence. For studies with small or large prevalence, near 0 or 1, the inverse variance method adds disproportionately large weight, variance becomes small, and the calculated CI may lie outside of the 0 to 1 range. Therefore, Freeman Tukey arcsine methodology is recommended to correct both variances instability and CIs [18].

Heterogeneity between studies was assessed by visual inspection of the forest plots, and by employing the I^2^ statistics and its related 95% CIs. The I^2^ values of 25, 50 and 75% was considered as low, medium and high heterogeneity, respectively [19]. A funnel plot symmetry was used to detect the presence of potential publication bias. Moreover, the Egger’s test was used to quantify the bias detected by the funnel plot [20]. Sensitivity analysis was done to determine the robustness of the results and evaluate the influence of single study on event estimate by omitting the individual study (leave out one approach). Sensitivity test eliminates each study step by step in the analysis to indicate the pooled effect sizes and related heterogeneity attributed by each individual study.

## Results

### Studies identification and retrieval

**Fig. 1:**
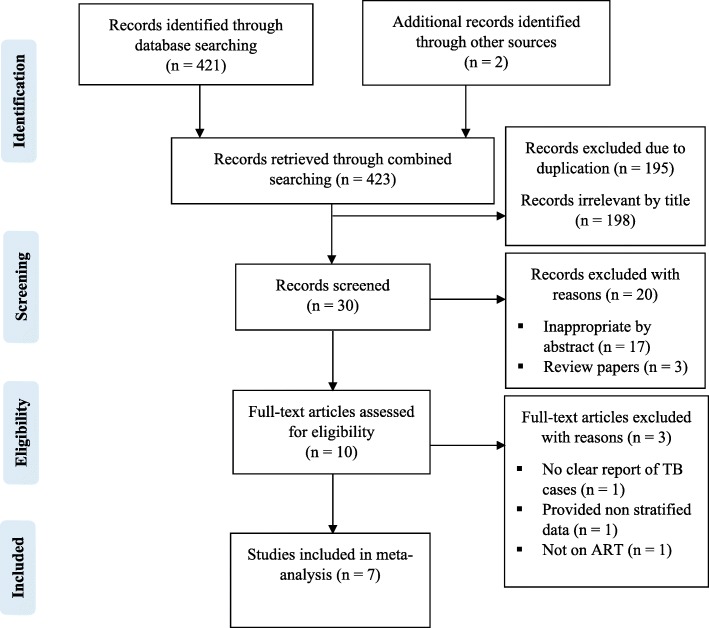
PRISMA flow chart for the studies screened, reviewed and included

The combined literature search strategy retrieved a total of 423 potential studies, of which 10 were screened for full text review and 7 studies were eligible to be included in the meta-analysis (Fig. 1).

### Characteristics of included studies

The articles included in the meta-analysis reported data from 2007 to 2015 and were published from 2014 to 2017. Among the included studies, four studies [21–24] were conducted in tertiary government hospitals whereas three studies [25–27] where conducted in both hospitals and the nearby health centers. The sample size of included studies ranged from 271 [21] to 5407 [24] subjects. A total sample size of 9838 participants were involved in the analysis. All studies included were retrospective cohort studies with a follow up duration for assessing the outcome ranging from 6 to 12 months. Consequently, five studies [21–25] determined the outcome after 6 months of IPT intervention while two studies [26, 27] assessed the outcome after 12 months of intervention. Besides, one study [25] administered IPT concomitantly with Cotrimoxazole (CPT) to the experimental group for 6 months. All studies provided IPT for intervention group for HIV positive patients receiving ART based on WHO recommendations [5]. Specifically, the intervention group received IPT for 6 to 12 months of duration at a dosage of 300 mg/day for adults and 5–10 mg/kg/day for children in all studies [28].

A total of 3769 participants were employed in the intervention group (receiving both ART and IPT), of whom 99 participants developed active TB. However, 6069 subjects were included in the control group (receiving only ART), and 668 of them developed active TB during the follow up period (Table 1).

**Table 1:** Characteristics of included studies (*n* = 7)

Study	Study area	Study design	Study period	Age (years)	Specimen	Methods employed	Participants	IPT Follow up in months	Experimental		Control	
									Events	Total	Events	Total
Abossie et al., 2017 [21]	Arba Minch, SNNP	RCS	Sep 2010 - Aug 2011	25–34	Sputum	Microscopy	271	6 months	12	138	37	133
Alemu et al., 2016 [25]	West Gojjam and South Gondar, Amhara	RCS	Sep 2009 -Sep 2014	1–15	Sputum	Microscopy, CXR and histopathology	645	6 months	11	119^a^	6	526
Nigussie et al., 2015 [22]	Addis Ababa	RCS	Aug 2014 - May2015	> 15	Sputum	Microscopy and CX	480	6 months	12	160	58	320
Edessa et al., 2014 [23]	Addis Ababa	RCS	Sep 2009 - Mar 2012	> 18	N/S	N/S	742	6 months	5	185	75	557
Yirdaw et al., 2014 [24]	Southern Ethiopia, SNNP	RCS	Sep 2007 - Aug 2010	0.16–82	Sputum	Microscopy and CXR	5407	6 months	51	2131	244	3276
Semu et al., 2017 [26]	Addis Ababa	RCS	2007 - Jan 2010	Adults	N/S	N/S	1842	12 months	4	942	71	900
Ahmed et al., 2015 [27]	North-eastern Ethiopia, Afar	RCS	July 2010 - May 2015	> 15	N/S	N/S	451	12 months	4	94	115	357

*Keys*: *CXR* Chest x-ray, *N/S* not specified, *RCS* Retrospective cohort study, *SNNP* Southern nations nationalities and peoples of Ethiopia, ^a^administered IPT concomitantly with Cotrimoxazole

### Synthesis of results

Given a considerable heterogeneity of the outcome across seven included studies, this meta-analysis has two outcomes: first, the protective effect of IPT on the incidence of active TB among HIV positive patients taking ART, and second, the incidence of active TB among HIV infected patients receiving ART with and without IPT within included studies.

### The effect of IPT on active TB incidence among HIV positive patients receiving ART

**Fig. 2:**
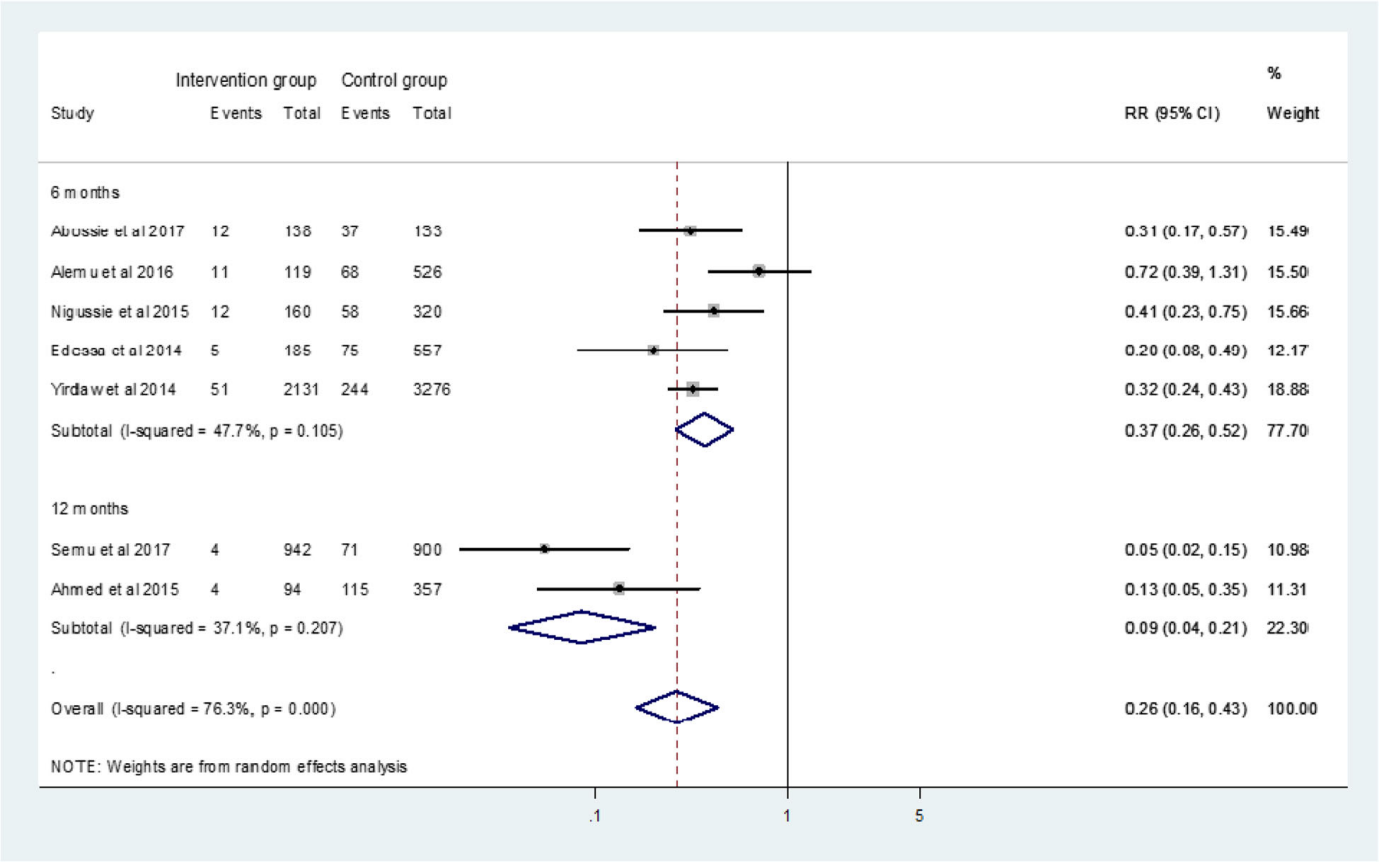
Forest plot for pooled protective effect estimate of IPT on active TB incidence among HIV positive patients receiving ART

The pooled effect of IPT in reducing incident TB among HIV infected patients taking ART was significant (RR: 0.26, 95% CI: 0.16 to 0.43) compared with no intervention group. Thus, IPT reduced the risk of active TB incidence by 74% in IPT group compared to no IPT intervention group. However, there is evidence of high statistical heterogeneity of outcome effects (I^2^ = 76.3%) within included studies. Besides, stratified analysis was done based on the duration of IPT intervention, and there was a significant change in the observed effect. Patients treated with IPT for 12 months have much more reduced risk of developing active TB (RR: 0.09, 95% CI: 0.04 to 0.21) compared to 6 months IPT intervention group (RR: 0.37, 95% CI: 0.26 to 0.52) (Fig. 2).

**Fig. 3:**
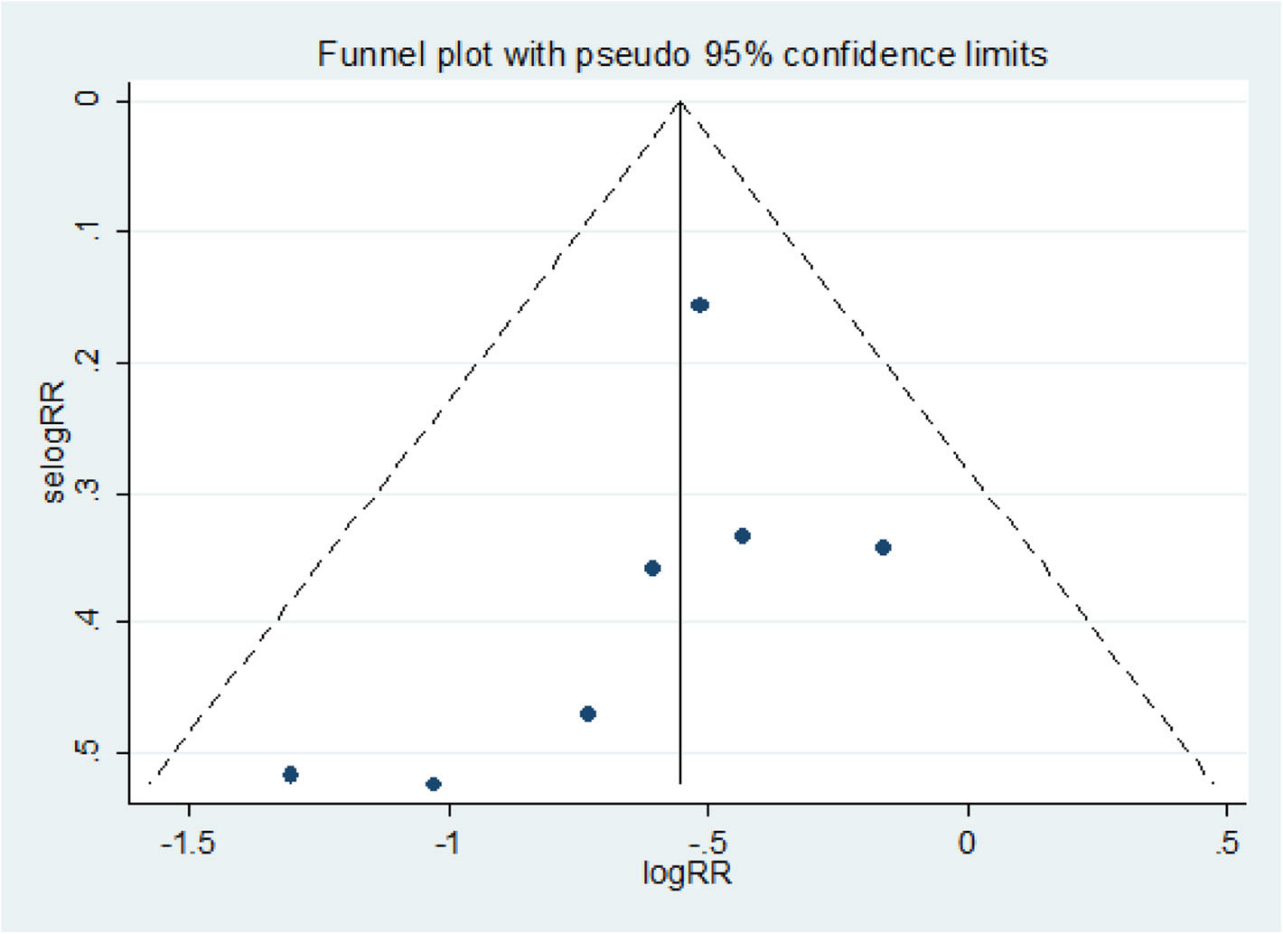
Funnel plot, evaluating the existence of publication bias for the analysis of risk ratio

As graphically demonstrated in the symmetrical funnel plot, there was no evidence of publication bias within included studies (Fig. 3). Moreover, this was also assured by Egger’s test (*p* = 0.27). The sensitivity analysis clearly demonstrated that the influence of individual studies on the summary effect estimate was not significant. Consequently, the pooled effect estimate of IPT on active TB incidence among HIV infected patients taking ART was steady and reliable when analyzed by omitting one study at a time (Table 2).

**Table 2:** Sensitivity analysis of IPT protective effect estimate on active TB incidence among HIV positive patients taking ART in Ethiopia

Study omitted	Estimate	95% CI
Abossie et al., 2017	0.25260973	0.1398754, 0.45620376
Alemu et al., 2016	0.22600834	0.13906604, 0.36730582
Nigussie et al., 2015	0.24055883	0.13436097, 0.43069464
Edessa et al., 2014	0.27287143	0.15840936, 0.47004056
Yirdaw et al., 2014	0.24392824	0.12219637, 0.48692921
Semu et al., 2017	0.33420488	0.23010728, 0.4853949
Ahmed et al., 2015	0.29158628	0.17552264, 0.48439652
Combined	0.26575699	0.16288678, 0.4335943

### Pooled incidence of active TB among HIV positive patients receiving ART

**Fig. 4:**
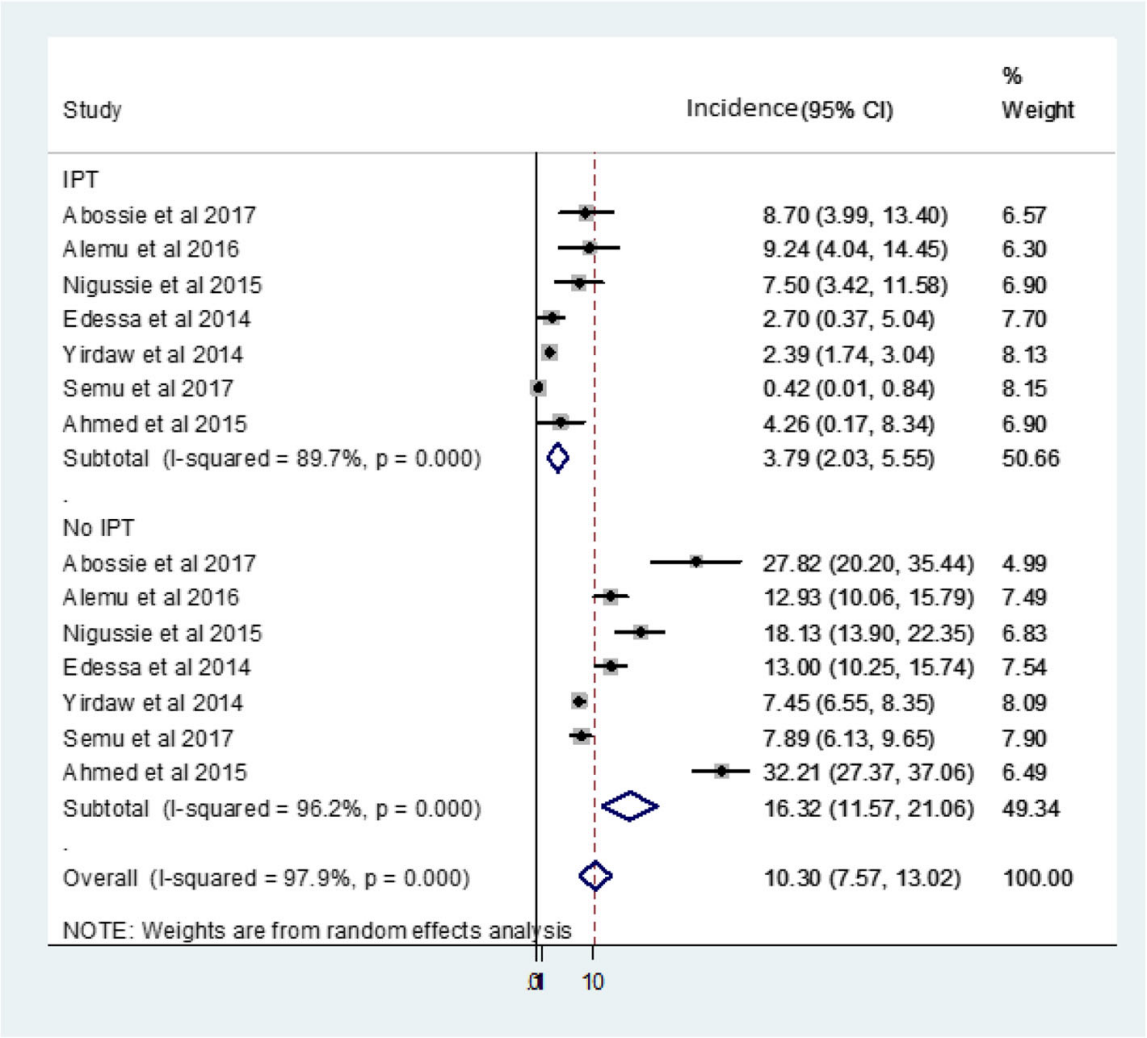
Forest plot indicating pooled incidence of active TB among HIV positive patients taking ART with and without IPT

The overall pooled incidence of active TB among HIV infected patients taking ART was 10.30 (95% CI; 7.57–13.02%, I^2^ = 97.9%, Egger’s test, *p* = 0.13). Subgroup analysis based on patients IPT status demonstrated that 3.79% (95% CI; 2.03–5.55%, I^2^ = 89.7%) and 16.32% (95% CI; 11.57–21.06%, I^2^ = 96.2%) TB incidence among HIV infected patients receiving ART with and without IPT intervention respectively (Fig. 4). There was no evidence of publication bias within included studies (Egger’s regression test p = 0.13). Sensitivity analysis revealed that the influence of individual studies on pooled incidence was insignificant (data not shown), indicating that the pooled incident TB was stable when evaluated by omitting one study at a time.

## Discussion

Although the advantage of TB preventive therapy is utmost in HIV infected patients with a positive TST, WHO recommended the intervention of IPT as a mainstay to reduce incident TB in people living with HIV regardless of TST status in high TB burden countries [29]. As per WHO recommendation, Ethiopia has also adopted and integrated it at the national HIV treatment guidelines [8]. Nevertheless, the number of HIV infected people receiving IPT in Ethiopia is limited due to different factors [10]. Thus, determining the effectiveness of IPT intervention as well as the relative incidence of TB in HIV positive patients taking ART with and without IPT is paramount important for proper IPT execution and management as well as to scale up the IPT program in high TB burden countries like Ethiopia.

Based on this meta-analysis, IPT reduced the incidence of active TB among HIV positive patients receiving ART significantly (RR: 0.26, 95% CI: 0.16 to 0.43). This was consistent with the report from Brazil, a 76% reduction in tuberculosis risk among HIV infected patients receiving both ART and IPT (adjusted relative hazard 0.24) [7]. Nevertheless, the pooled effect estimate of IPT in reducing TB incidence in this meta-analysis was slightly lower than the reports from South Africa (RR: 0.11, 95% CI: 0.04 to 0.32), 89% reduction in TB risk [30]. This could be due to IPT might be administered for extended periods in South Africa or patients may have better adherence rate to IPT compared to Ethiopian counterparts.

Furthermore, subgroup analysis indicated that IPT for 12 months has reduced the risk of active TB incidence by 91% (RR: 0.09, 95% CI: 0.04 to 0.21) compared to 6 months IPT intervention which has a 63% reduction in TB risk (RR: 0.37, 95% CI: 0.26 to 0.52). Therefore, 12 months of IPT was more efficacious in reducing active TB incidence than 6 months of IPT intervention among HIV positive patients taking ART. As a result, extended IPT intervention might benefit through different mechanisms. First, it might improve eradication of latent tuberculosis that was present at enrolment. Second, it may potentially prevent new infections for longer time than short-term INH preventive treatment that may have a durable protective effect. Finally, it might be partly due to as time goes after ART initiation, the synergetic effect of ART and IPT in reducing active TB incidence is increased as a result of CD4^+^ T cells recovery and viral load suppression.

Besides, the pooled incidence of active TB among HIV infected patients receiving ART within included studies was assessed. Accordingly, the overall pooled TB incidence was 10.30 (95% CI; 7.57–13.02%). However, previous studies showed that 14.27% [31], 4.4% [32] and 3.41% [33] in India, Tanzania and South Africa respectively. In addition, subgroup analysis showed that 3.79% (95% CI; 2.03–5.55%) and 16.32% (95% CI; 11.57–21.06%) pooled incident TB among HIV infected patients receiving ART with and without IPT respectively. This was in line with the previous report, 3.8% [34] and 17% incident TB [35] among HIV positive patients receiving ART with and without IPT respectively. However, lower TB incidence in IPT group was reported from Cape Town, South Africa, 2.3% [6] and Rio de Janeiro, Brazil 0.8% [7] compared to this study. On the other hand, lower incident TB among HIV infected patients receiving ART without IPT was reported from Johannesburg, South Africa 6.65% [36] and comparatively higher TB was stated in Nepal 27.3% [37] compared to the present findings. The variance in incident TB among HIV infected patients taking ART might be due to differences in TB endemicity, adherence to ART, socioeconomic factors and degree of immune suppression in the studied cohorts in these different countries. Moreover, the difference in participants’ IPT adherence may also partly contribute to the discrepancy of incident TB reported among HIV infected patients receiving ART with IPT in these different countries.

### Limitations

Even if this study has shown the translational highway to integrating and implementing IPT in HIV care and treatment management guidelines in Ethiopian settings, it has some limitations to be considered. First, the number of included studies were limited (only 7). Second, tuberculin skin test (TST) status was not determined before IPT initiation. Third, the emergence of INH resistant TB is not reported. Forth, INH-ART drug interaction induced toxicities were not reported in the included studies which may be one of the hindrances to IPT implementation in the country. Fifth, given the difficulty of diagnosing TB in HIV infected persons, the specimen used and methods employed to diagnose TB were not specified for three included studies. Lastly, all included studies in this analysis were retrospective cohort studies, which share the limitation of retrospective study designs.

## Conclusions

This meta-analysis indicated that IPT was effective in reducing incident TB among HIV positive patients receiving ART. Besides, the duration of IPT has a significant effect on its protective role. Moreover, this study also sought high incidence of active TB among HIV infected patients receiving ART without IPT compared to IPT intervention group. Thus, scaling up the IPT program and its strict compliance is necessary to overcome the rapidly growing HIV associated incident TB in Ethiopia.

## Additional files

Additional file 1: Prisma 2009 Checklist. (DOC 70 kb)

Additional file 2: Quality assessment for included studies (Newcastle-Ottawa quality assessment scale). (DOCX 13 kb)

## Abbreviations

ART: Antiretroviral therapy
CI: Confidence interval
CPT: Cotrimoxazole
HAART: highly active antiretroviral therapy
HIV: Human immunodeficiency virus
INH: Isoniazid
IPI: Isoniazid preventive therapy
PLWH: people living with HIV
RR: Risk ratio
TB: Tuberculosis
TST: tuberculin skin test
WHO: World health organization
