# Supplementary material for: The protective effect of isoniazid preventive therapy on tuberculosis incidence among HIV positive patients receiving ART in Ethiopian settings: a meta-analysis
Source: BMC Infect Dis. 2019 May 10;19:405. doi: 10.1186/s12879-019-4031-2 (PMC6511123; doi:10.1186/s12879-019-4031-2)
Supplement: Supplementary file 2 — Quality assessment for included studies (Newcastle-Ottawa quality assessment scale). (DOCX 13 kb) [file 12879_2019_4031_MOESM2_ESM.docx]

**Additional file 1**

Quality assessment for included studies (Newcastle-Ottawa quality assessment scale)

| Study | Selection | Comparability | Outcome/Exposure |
| --- | --- | --- | --- |
| Abossie et al, 2017 | * * * * | * * | * * * |
| Alemu et al, 2016 | * * * * | * * | * * * |
| Nigussie et al, 2015 | * * * * | * * | * * * |
| Edessa et al, 2014 | * * * | * | * * |
| Yirdaw et al, 2014 | * * * * | * * | * * * |
| Semu et al, 2017 | * * * | * | * * |
| Ahmed et al, 2015 | * * * | * | * * |
